# Research Priorities to End the Adolescent HIV Epidemic in the United States: Viewpoint

**DOI:** 10.2196/22279

**Published:** 2021-01-04

**Authors:** M Isabel Fernandez, Gary W Harper, Lisa B Hightow-Weidman, Bill G Kapogiannis, Kenneth H Mayer, Jeffrey T Parsons, Mary Jane Rotheram-Borus, Arlene C Seña, Patrick S Sullivan

**Affiliations:** 1 College of Osteopathic Medicine Nova Southeastern University Fort Lauderdale, FL United States; 2 Department of Health Behavior and Health Education School of Public Health University of Michigan Ann Arbor, MI United States; 3 Institute for Global Health and Infectious Diseases University of North Carolina at Chapel Hill Chapel Hill, NC United States; 4 Maternal and Pediatric Infectious Disease Branch Eunice Kennedy Shriver National Institute of Child Health and Human Development Bethesda, MD United States; 5 The Fenway Institute Fenway Health Boston, MA United States; 6 Mindful Designs Teaneck, NJ United States; 7 Department of Psychiatry University of California, Los Angeles Los Angeles, CA United States; 8 Department of Epidemiology Rollins School of Public Health Emory University Atlanta, GA United States

**Keywords:** HIV/AIDS, adolescents

## Abstract

Youth represent 21% of new HIV diagnoses in the United States. Gay, bisexual, and transgender (GBT) youth, particularly those from communities of color, and youth who are homeless, incarcerated, in institutional settings, or engaging in transactional sex are most greatly impacted. Compared with adults, youth have lower levels of HIV serostatus awareness, uptake of antiretroviral therapy (ART), and adherence. Widespread availability of ART has revolutionized prevention and treatment for both youth at high risk for HIV acquisition and youth living with HIV, increasing the need to integrate behavioral interventions with biomedical strategies. The investigators of the Adolescent Medicine Trials Network for HIV/AIDS Interventions (ATN) completed a research prioritization process in 2019, focusing on research gaps to be addressed to effectively control HIV spread among American youth. The investigators prioritized research in the following areas: (1) innovative interventions for youth to increase screening, uptake, engagement, and retention in HIV prevention (eg, pre-exposure prophylaxis) and treatment services; (2) structural changes in health systems to facilitate routine delivery of HIV services; (3) biomedical strategies to increase ART impact, prevent HIV transmission, and cure HIV; (4) mobile technologies to reduce implementation costs and increase acceptability of HIV interventions; and (5) data-informed policies to reduce HIV-related disparities and increase support and services for GBT youth and youth living with HIV. ATN’s research priorities provide a roadmap for addressing the HIV epidemic among youth. To reach this goal, researchers, policy makers, and health care providers must work together to develop, test, and disseminate novel biobehavioral interventions for youth.

## Introduction

### The HIV Epidemic in the United States

HIV infections among adolescents and young adults aged 13 to 24 years have more than doubled in the last 15 years, and these individuals represented 21% of the epidemic population (approximately 7100 infected youth) in the United States in 2018 [1-3]. Gay, bisexual, and transgender (GBT) youth represented up to 70% of new infections among youth in 2017. Among GBT youth, 37% were Black youth and 29% were Hispanic/Latino youth in terms of race/ethnicity, whereas 28% were White youth [3]. HIV also disproportionally impacts other marginalized youth, including those who are homeless, those who are incarcerated, those placed in foster care or other types of institutions, and those who engage in transactional sex [4-18]. Furthermore, there are geographic disparities in new HIV diagnoses. As reflected in the Ending the HIV Epidemic Initiative, southern states accounted for 52% of new HIV diagnoses in 2017 [19]. To stop HIV among adolescents, it is essential to routinely engage youth at the highest risk for HIV in prevention strategies and to achieve and sustain viral suppression in all youth living with HIV.

### HIV Prevention and Treatment Continua

There are sequenced steps, referred to as the HIV treatment continuum and the HIV prevention continuum, that provide a metric for monitoring success toward ending the HIV epidemic. The first step in the HIV treatment continuum is to identify youth living with undiagnosed HIV infection. To achieve this, youth at high risk for HIV acquisition need to be tested for HIV every 3 months. However, 60% of youth living with HIV remain undiagnosed, and this is the highest rate in any age group [2]. Once diagnosed, the next steps are to ensure that youth living with HIV are linked to care, receive antiretroviral therapy (ART), and are regularly monitored so they can achieve viral suppression. ART, which is more effective and easier to take than before, improves quality of life and reduces early mortality. Furthermore, when youth achieve viral suppression, the risk of HIV transmission is reduced [9,10]. Impressively, about two-thirds (68%) of youth living with HIV are linked to care within 1 month of their diagnosis [13]. Among youth in care, 98% were prescribed antiretroviral therapy and 89% achieved viral suppression at 1 year [13]. However, less than half (34%-44%) of youth living with HIV are retained in HIV care for a year or more, and viral suppression among youth living with HIV is only about 16% at 5 years after diagnosis (compared with 58% among adults) [14,15], indicating that linkage to care is insufficient to achieve and sustain viral suppression [20].

Similar to the HIV treatment continuum, the HIV prevention continuum involves several key steps [21]. These include (1) access to health care; (2) regular HIV testing for youth at high risk, especially among GBT youth; (3) adoption of prevention strategies, such as condom use, pre-exposure prophylaxis (PrEP), and postexposure prophylaxis (PEP) for one time acute exposure; and (4) retention and adherence to these prevention strategies over time. Many youth, especially GBT youth, have limited access to health care. Approximately one in three GBT youth between 12 and 17 years of age do not receive preventive care, and one in three do not seek or receive care within a year of symptom onset for a health problem [16,18,22]. Youth who need PrEP are those least aware of and least likely to use PrEP [17,18]. Despite the demonstration of PrEP acceptability among GBT youth [23], PrEP utilization estimated from 2017 national data was only 9.4% among young men who have sex with men (MSM) compared with 19.9% among all MSM [24]. Although the proportion of health care providers prescribing PrEP in the United States has risen to 24%, differences in PrEP use between racial/ethnic groups remain, with a use percentage of 26% among eligible Black MSM versus 42% among White MSM [25]. Moreover, once initiated, PrEP adherence has been found to be suboptimal among young MSM, even when paired with tailored behavioral interventions [23,26].

In light of the suboptimal utilization of the treatment and prevention continua, the Adolescent Medicine Trials Network for HIV/AIDS Interventions (ATN) identified research gaps and defined five priority areas to guide its research agenda. The ATN is the only domestic research network (established in 2001) focused on youth at high risk for HIV and youth living with HIV (age 12-24 years). In this paper, we outline ATN’s research agenda for reducing new HIV infections among youth in the United States.

## Overview of the ATN

**Figure 1:**
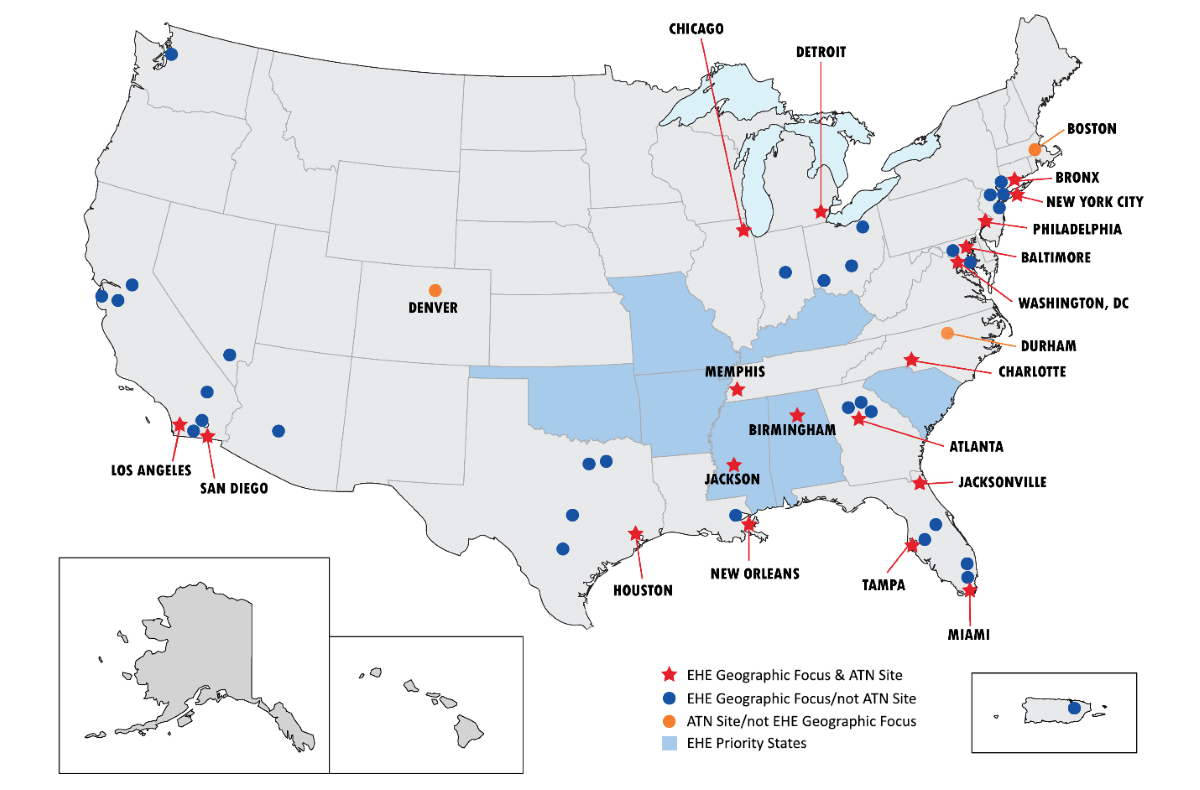
Map of Adolescent Medicine Trials Network for HIV/AIDS Interventions study sites and Ending the HIV Epidemic geographic areas.

The ATN is a collaborative National Institutes of Health (NIH)–supported research network with the goal of reducing the number of HIV infections among youth by (1) increasing the number of youth who know their HIV status; (2) developing, testing, and scaling up sustained use of biobehavioral approaches (condoms, PrEP, and PEP) among youth at high risk for HIV; and (3) increasing the number of youth living with HIV who achieve and sustain viral suppression. The *Eunice Kennedy Shriver* National Institute of Child Health and Human Development provides the primary financial support for this network; additional resources are provided by the National Institute on Drug Abuse, the National Institute of Mental Health, and the National Institute on Minority Health and Health Disparities. As shown in Figure 1, ATN sites are located in heavily impacted areas, many of which were identified in the Ending the HIV Epidemic: A Plan for America (EHE) initiative [27]. These sites reflect the concentration and the distribution of youth living with HIV nationally.

First established in 2001, the ATN is in its fourth project period. Currently, the ATN is comprised of three thematically focused peer-reviewed Research Program Grants (U19) (Scale It Up, CARES, and iTech), a Coordinating Center (U24), a number of independent and cross-network research protocols managed by the U24, a Diversity Scholars Program, and several cross-network working groups. The ATN facilitates the sharing of ideas, data, scientific expertise, and specialized resources, such as equipment, services, and clinical facilities. Although all ATN protocols address one or more steps in the HIV treatment and/or prevention continua, they are addressed through the thematic lens of their managing U19. Thus, research projects are complementary and act synergistically to achieve the network’s goals (a list of currently funded ATN projects can be found on the ATN website) [28]. A brief summary of each U19 is provided below.

ATN Scale It Up includes six studies specifically focused on implementation science and the process of improving self-management among youth [29]. This multisite U19 identifies and builds on efficacious and effective interventions to improve self-management among youth at high risk for HIV infection and youth living with HIV. One of its goals is to develop, test, and disseminate new methods for implementation and analysis with strong theoretical foundations, such as tailored motivational interviewing to help adolescent medicine providers advance the movement of their patients across the treatment and care continua. To attain this goal across all the interventions being tested in Scale It Up, researchers are assessing how youth self-management varies over time, is improved by interventions, and mediates intervention effects.

CARES is focused on identifying and leveraging novel community-based settings to act as gateways where youth at high risk and youth living with HIV can be engaged in HIV prevention and treatment [30]. Youth recruited into one of CARES’ three studies complete a behavioral assessment and are tested for sexually transmitted infections (STIs) every 4 months. Both seronegative youth and youth living with HIV enrolled in the studies receive a stepped care model of increasingly intensive interventions, which include text messaging and mobile self-assessment, peer support through social media groups, and interpersonal coaching. This stepped care model has been successfully used with other chronic diseases [31-33], and CARES is evaluating the model’s efficacy for HIV prevention and treatment.

iTech aims to lower the burden of HIV infection by developing and evaluating innovative interdisciplinary research on technology-based interventions across the HIV prevention and care continua for youth at high risk or youth living with HIV [34]. Each of iTech’s 11 research projects includes the use of technological innovations (eg, apps/mobile websites, videoconferencing/telehealth, and home-based HIV/STI testing) to advance the field. The iTech objectives are discussed in more detail under the Technology section below.

The Coordinating Center provides support, coordination, and operational infrastructure to the three ATN research programs and provides expertise in project management, data management, and statistics to the ATN [35]. The Coordinating Center manages multiple network studies, collaborating with principal investigators and project sites at health care organizations and universities around the country.

## Development of the ATN Research Priorities

**Figure 2:**
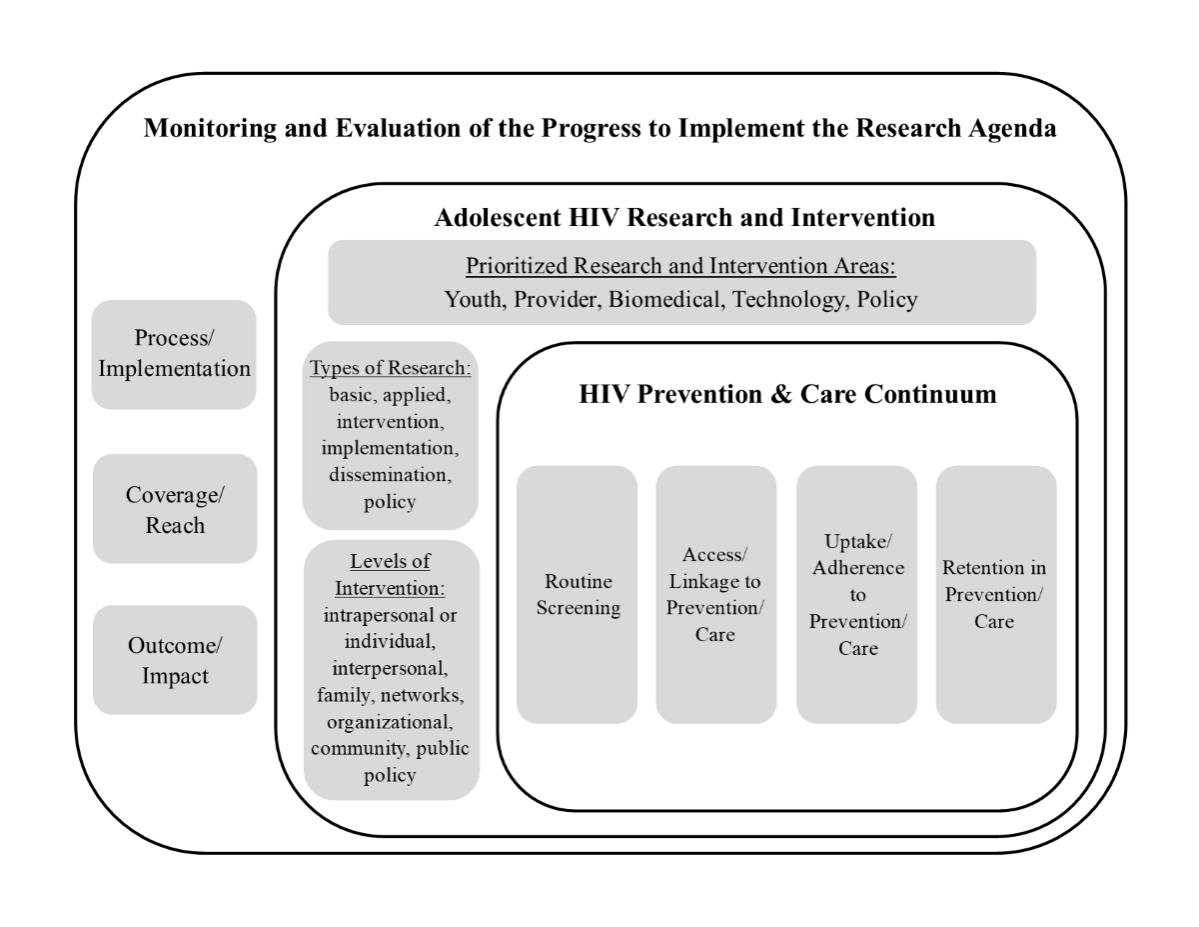
A nested model of the research needed to defeat the rising HIV epidemic among adolescents and young adults in the United States. This model includes the primary targeted outcomes to achieve reductions in HIV among youth at high risk and viral suppression among youth living with HIV.

The Executive Committee (EC), the leadership and governing body of the ATN, is comprised of representatives from each U19, the Coordinating Center, participating National Institutes of Health, selected scientific experts, and community youth representatives. The EC identifies emergent scientific priorities, defines the research agenda, and fosters collaboration with other HIV research networks and investigators. To begin the research prioritization process, the EC held a series of conference calls, workshops, presentations, and meetings. Furthermore, the EC surveyed ATN investigators and consulted with scientific experts outside of the EC to identify the key research issues, priorities, and knowledge gaps regarding the adolescent HIV/AIDS epidemic in the country. The EC completed the research prioritization process in 2019 and identified the following five priority research areas: (1) innovative strategies to engage youth in HIV treatment and prevention, (2) health systems and provider interventions, (3) biomedical interventions, (4) technology, and (5) data-informed policies. A nested model (Figure 2) was also created to illustrate the complex set of factors that need to be considered in attempting to implement and evaluate the newly developed research plan.

At the core of the nested model lies the continua of care elements for both HIV prevention and HIV treatment, since these are the focal points for ATN’s research and intervention work. At the next level are our prioritized research areas that could be applied to all of the factors within the continua of care. Also included in this level are descriptions of the various types of research that could be conducted in order to explore prioritized research areas (ie, basic, applied, intervention, implementation, dissemination, and policy) and the levels at which ATN’s research and interventions could be focused (ie, intrapersonal or individual, interpersonal, family, networks, organizational, community, and public policy). The outermost level demonstrates the need to continually monitor and evaluate the research agenda to assess its utility and to offer insights for needed adjustments. We suggest that such monitoring and evaluation should occur at three different levels in order to ensure maximum benefit to adolescents, including process/implementation, coverage/reach, and outcome/impact.

Three principles emerged that guided our thinking as we developed the research priorities. First, the recognition that segmenting by serostatus was no longer salient since the approaches (eg, sustained health care engagement and ART adherence for PrEP or treatment as prevention) needed to promote movement along the HIV treatment continuum for youth living with HIV and the HIV prevention continuum for youth at risk are similar. Second, at each step on these continua, youth often confront multiple barriers to care that may stem from personal and interpersonal issues, societal/community norms, structural factors, and challenges with the health care system. Furthermore, the salience of these barriers varies by geography, context, and sociocultural contexts. Third, there are concurrent developmental processes unfolding during adolescence. Youth are most likely to first initiate risky behaviors that lead to HIV acquisition or transmission during adolescence, including sexual activity without a condom, and alcohol and drug use [36-38]. Adolescence is also the period of onset of many lifelong mental health challenges, particularly among sexual, gender, racial, and ethnic minority youth [39]. These developmental trajectories offer opportunities for interventions, such as comprehensive sex and substance abuse prevention, and age-appropriate and culturally sensitive HIV-prevention programs [40]. The developmental processes are more complicated for GBT youth, who face additional challenges stemming from their marginalized and stigmatized sexual orientation [41-44]. For instance, unlike their heterosexual peers, GBT youth must decide who, when, how, where, and what to disclose regarding their sexual orientation and/or their HIV status [45,46].

## ATN’s Priority Research Areas

### Overview

In this section, we describe each research priority area and highlight some of ATN’s efforts to address them. It is important to note that the ATN regularly reviews these research priorities to ensure that they continue to be responsive to scientific advances, changes in the epidemic of HIV, and emerging needs in the youth we serve.

### Innovative Strategies to Engage Youth in HIV Treatment and Prevention

Engaging youth in interventions to increase their uptake, adherence, and retention with HIV treatment and prevention services poses great challenges but remains a critical research priority. It has long been known that community agencies and venues where youth congregate are fruitful settings for engaging youth [47,48]. Providing safe and affirming spaces where youth can receive culturally congruent support and services can promote engagement of HIV prevention and treatment services [49]. The importance of using text messages and social media platforms and apps for engaging and retaining youth is increasing [50-54]. Recent community mobilization programs, focused on addressing structural-level factors to improve HIV testing and prevention, yielded mixed results [55-57].

Leveraging and expanding this knowledge, ATN investigators have launched innovative studies that are examining social media–based strategies for engaging, recruiting, and retaining youth in research studies and testing technology-based interventions. For example, one current ATN project focuses on helping youth living with HIV secure jobs through vocational training to overcome socioeconomic barriers to health care [58]. ATN studies are focusing on how to more effectively reach youth with adaptations or novel delivery strategies of evidence-based interventions that are lower cost, more attractive, and more accessible, while having a greater impact on decreasing HIV transmission and acquisition, and increasing engagement and adherence to therapeutic and preventive interventions.

Implementation science research is one of the most important approaches for optimizing interventions and maximizing their impact on youth at high risk for HIV acquisition and youth living with HIV. The ATN employs innovative methods, such as implementation-effectiveness hybrids, to address effectiveness and implementation along the same timeline [59]. The portfolio of current ATN studies also includes interventions that were designed with dissemination in mind. They can be scaled rapidly, reduce the need to precisely replicate a manualized intervention, and focus on each youth’s personal risks, tailoring the work to address the variability that occurs across and between communities [60,61]. The studies also build on evidence-based interventions systematically assembled and disseminated by the Centers for Disease Control and Prevention (CDC), Substance Abuse and Mental Health Services Administration (SAMHSA), and Health Resources and Services Administration (HRSA) to identify the most robust intervention components for wider utilization and scale-up.

### Health Systems and Provider Interventions

Although researchers have identified that adolescents prefer medical care that is local, integrated, quick, confidential, nonprejudicial, hassle-free, and inexpensive [62,63], this type of care is yet to become widely available. Lesbian, gay, bisexual, transgender, and queer (LGBTQ) youth face particular challenges in accessing and receiving culturally affirming health care services, as health care providers and others with whom youth interact in care settings may hold stigmatizing views of LGBTQ youth [64-66]. Such forms of stigma perpetuated by health care providers and others in clinical settings have been shown to be barriers to seeking health care, especially among transgender young people [67-69]. These challenges may be exacerbated for LGBTQ youth of color, as racism is a form of intersectional stigma that negatively impacts access to health care, especially for Black LGBTQ youth [65,70,71]. Clinical providers can play a pivotal role in promoting change within their organization, reducing stigma, and increasing youths’ engagement and progression through the HIV prevention and care continua. Health system and provider interventions can promote changes in practice as well as in care settings. For example, training medical, clerical, and administrative staff to be culturally responsive to GBT youth clients successfully transformed policies and protocols of a set of health care agencies [72]. Providing feedback on the perceived quality of a clinic’s care and services can also improve the quality of care and the services provided. For example, a current ATN project in iTech sends “mystery shoppers,” adolescent consumer advocates, to visit existing HIV testing sites to evaluate their HIV counseling and testing services according to a number of youth-focused dimensions. Testing sites receive informational reports that they can use to improve service delivery [73].

Despite the efficacy of PrEP and PEP in reducing HIV acquisition, some providers are reticent to prescribe these medications to youth because they are concerned about risk compensation (ie, adjusting one’s behavior according to the perceived level of risk), nonadherence, and ART toxicities [74,75]. Other providers have expressed concerns regarding prescribing PrEP and ART to substance users [76]. Clearly, provider-based interventions to promote PrEP and PEP are needed. Although several approaches for scaling up PrEP and PEP in youth have been developed [77,78], they are yet to be widely disseminated and some are yet to be rigorously evaluated. For instance, public health jurisdictions, such as the New York City Health Department, are using academic detailing, a strategy typically used by pharmaceutical companies that engages providers in their work setting, to efficiently educate providers about PrEP [79,80]. Interventions to help providers to deliver culturally affirming care to GBT youth are also needed. Although there are many text and web resources to promote the use of PrEP among primary care providers, the efficacy of these programs to improve practice and services for youth has not been fully evaluated [81-83]. TMI, one of the studies in Scale it Up, addresses this need.

### Biomedical Interventions

PrEP, PEP, and treatment as prevention have become important biomedical interventions that can either protect youth from becoming infected or ensure longer life and higher quality of life for youth living with HIV [9,84,85]. The first trials of PrEP (tenofovir plus emtricitabine) in 15- to 21-year-old youth at high risk for HIV infection, conducted by the ATN, demonstrated that PrEP offered high levels of protection for youth who were adherent and did not identify safety concerns [26,86,87]. Concerns related to long-term adherence to daily oral therapy has led researchers to examine the efficacy of long-acting antiretroviral agents, particularly injectable formulations of ART, which are currently being evaluated in phase III studies with adults, and broadly neutralizing antibodies, which are currently in phase IIb testing [88-90]. To help ensure that these novel formulations are approved for youth, the ATN is collaborating with the HIV Prevention Trials Network (HPTN) to examine the acceptability, tolerability, and adherence to injectable cabotegravir in youth. Because of its experience navigating the ethical, legal, and regulatory issues associated with the participation of youth in trials of biomedical interventions, the ATN has and will continue to play a pivotal role in advancing biomedical prevention efforts for youth [91,92].

HIV vaccine research and the development of immune-based and topical microbicidal–based therapies are other promising biomedical interventions [93-97]. Basic and clinical scientists have made major developments in knowledge about HIV reservoirs and interventions for an HIV cure, although attempts to achieve a cure have been unsuccessful to date [98,99]. One of the studies in the CARES U19 focuses on treatment of acutely infected youth, comparing their viral reservoirs over time to those of youth with established infections who were treatment naive. This project will provide important data on developmentally linked immunological processes associated with early and sustained viral suppression. Additionally, there has been an improved understanding of the role of the mucosal microbiome, semen exposure, and STIs in the risk of HIV acquisition and transmission [100]. The most futuristic approaches are studies of gene editing, which look to either eliminate the *CCR5* gene or mutate the HIV virus successively so that it is no longer robust and easily transferable [101]. When these interventions are ready for testing, the ATN is well positioned to initiate trials of these biomedical studies in collaboration with researchers conducting trials with adults.

### Technology

Technology use is ubiquitous among adolescents; thus, its power and reach can be harnessed for interventions targeting each stage of the prevention and care continua. Technology offers unique opportunities to recruit, engage, and retain adolescents in research through provision of tailored messages, inclusion of game-based elements, and delivery of personalized theory-based intervention components and health content [102]. Given that more than 96% of youth aged 18 to 29 years in the United States have smartphones and regularly use the internet for a variety of activities, interventions delivered through the internet and mobile phones are highly acceptable to youth [103,104]. Although the costs for initial start-up and development of technology-based interventions are high, once developed, the associated costs of adaptation and dissemination are more moderate. It is not surprising that the number of researchers developing and testing internet- and app-based HIV interventions is rapidly increasing. Unfortunately, these efforts are not well coordinated, and there is a proliferation of similar apps or websites that fail to make novel contributions or address unanswered questions [105,106].

Each ATN U19 program (iTech, CARES, and Scale it Up) is developing and evaluating innovative technology–based interventions across the HIV prevention and care continua. iTech focuses on the evaluation of technologically oriented and youth-oriented interventions, with each project utilizing mobile technologies, including apps, mobile-optimized websites, and online video counseling. The apps within iTech are tailored to the population addressed (eg, seronegative youth and youth living with HIV), the targeted health outcome (eg, PrEP uptake or adherence, engagement with health care, and viral suppression), and the technology platform utilized. iTech will foster the identification of the specific features across apps that are most used and most useful for specific populations. Using a harmonized set of technology-focused metrics and paradata for evaluation and cost-effectiveness analyses, iTech represents a coordinated effort that will allow for more impactful technology-based interventions. For example, one of its projects is a three-arm, randomized, controlled trial that is testing the efficacy of P3 (Prepared, Protected, emPowered), a novel, theory-based mobile app to promote PrEP adherence through social networking and gamification. This app utilizes game mechanics (rewards and unlocking app features) and social networking/peer support to also improve retention in PrEP clinical care and PrEP persistence among GBT youth [107].

In contrast, CARES adopted low-cost, scalable, device-agnostic platforms that are utilized across all subpopulations and outcomes, with a focus on personalizing risk messages within platforms. Personalized messaging will be the same regardless of the delivery format (text message, phone call, and video chat). CARES interventions combine text messages and peer-support chat rooms with more traditional counseling methods. The CARES approach focuses on messages and probes, which address the following core functions of behavioral change: establish a framework for change, convey necessary information, build self-management skills, address barriers, and provide tools and support. Scale It Up is conducting an effectiveness trial, called SMART, of an intervention comparing text messaging and cell phone support to improve HIV medication adherence among youth living with HIV. Youth are recruited and enrolled online, using social media and app-based approaches. Across all ATN projects, the opportunity to provide a tailored suite of technology-based tools is a useful and innovative way to engage youth at high risk or youth living with HIV to improve HIV-related prevention and treatment outcomes (Table 1).

**Table 1:** Technology-based tools utilized in ATN studies.

Name of the study	Brief description of technology-based tools	Associated theories	Targeted outcomes	Research program
YouThrive	Web app including peer support via social networking, daily tips for living with HIV, self-monitoring tools, and goal setting/tracking	Information, motivation, and behavioral skills model	Viral suppression	iTech
Get Connected	Web app with content tailored to demographic characteristics, HIV/STI^a^ risk behaviors, and sociocultural contexts of individual participants	Integrated behavior model and self-determination theory	Uptake of HIV prevention services, PrEP^b^ awareness, and willingness	iTech
LYNX	Mobile app including personalized HIV risk scores, HIV/STI testing, and prevention information	Information, motivation, and behavioral skills model	HIV/STI testing and PrEP uptake	iTech
My Choices	Mobile app including tools for tracking HIV risk, HIV/STI testing, and prevention information	Social cognitive theory	HIV/STI testing and PrEP uptake	iTech
P3	Mobile app utilizing game mechanics and social networking features P3+ includes provision of in-app adherence coaching	Social cognitive theory and Fogg behavioral model	PrEP adherence, retention in PrEP care, and PrEP persistence	iTech
SMART	Comparison of text messaging–based and cell phone–based adherence support	Supportive accountability model	Viral suppression and adherence self-management	Scale It Up
TMI	Automatic feedback reports to providers to improve motivational interviewing competency	Modeling, rehearsal (verbal/behavioral), and feedback strategies	Motivational interviewing competency	Scale It Up
Stepped Care for Youth	Stepped care intervention utilizing text messaging and monitoring, peer support via online social networks, and e-coaching	N/A^c^	Viral suppression	CARES
Engaging Seronegative Youth	Text messaging and monitoring, peer support via online social networks, and e-coaching	N/A	HIV/STI testing, PrEP uptake, and adherence	CARES
Triggered Escalating Real-time Adherence (TERA)	Electronic dose monitoring, adherence monitoring via text messaging, and e-coaching as needed	Therapeutic drug monitoring	ART adherence and viral suppression	Coordinating Center
ePrEP	Mobile app–based home care system for PrEP prescription and care	Anderson behavioral model adapted to HIV care	PrEP adherence, retention in PrEP care, and PrEP persistence	iTech
TechStep	Stepped care intervention utilizing text messaging, a web app, and e-coaching	Information, motivation, and behavioral skills model	PrEP uptake and reduction of HIV risk behaviors	iTech

^a^STI: sexually transmitted infection.

^b^PrEP: pre-exposure prophylaxis.

^c^N/A: not applicable.

### Data-Informed Policies

Actionable policies are optimally derived from and shaped by data. Fortunately, many of the recent state and federal policy shifts in the United States reflect the belief that youth should be empowered to advocate and take individual responsibility for their health. Some of these shifts include expanding minors’ authority to consent to health care, including care related to sexual activity and STIs, HIV testing and treatment, and the constitutional right to privacy regarding a minor’s decision to obtain contraceptives [108].

Most states today have a policy requiring some form of HIV education. Federally funded school-based HIV prevention programs typically do not address issues related to GBT youth and thus are not relevant for these youth [109,110]. For instance, many state-supported abstinence-only programs exclude discussions of same-gender sexuality (eg, State Abstinence Education Grant Program). Furthermore, the efficacy of such programs to reduce STI incidence and HIV prevalence has not been established since the majority of evaluations were not powered to detect changes in biological outcomes. A recent meta-analysis of school-based interventions reported that only one of seven programs reduced STI incidence and none decreased HIV incidence [111]. Thus, rigorous evaluation of multilevel school-based programs is being considered as a future ATN research priority.

Evidence on the relationship between school climate and hate crimes should be used to inform and create policies to monitor the increase and levy consequences for discriminatory acts against marginalized groups such as GBT youth. Similarly, policies to create safe spaces for GBT youth and monitor the success of engaging GBT youth and other youth at high risk for HIV acquisition should also be enacted [112]. There is evidence that in jurisdictions with more affirming LGBTQ school climates, these youth reported fewer days of episodic drinking and fewer drinking days at school [113]. The ATN is gathering and disseminating data to policy makers, public health officials, and other officials to make informed decisions and implement efficacious HIV prevention and treatment models for US youth. The Community-Engaged Dissemination and Implementation Research (CEDI) Workgroup is working in collaboration with ATN members to translate their evidence-based research findings for dissemination and implementation in clinic and community settings, and to inform policy and advocacy for delivery of care. Another example is the work being conducted by the ATN Modeling Core, which is developing a detailed health policy mathematical model to monitor and evaluate HIV in adolescents and young adults. The Modeling Core is using a novel approach to microsimulation modeling of HIV disease progression, patterns of care, and treatment outcomes, and applying innovative statistical methods to populate the model with data about patterns of health care, HIV viral load trajectories, and ART derived from completed ATN studies and other national studies. This model will be used to evaluate the potential clinical and economic impacts of ATN intervention trials to support medication adherence, retention in care, and improved clinical outcomes for youth living with HIV and inform decision makers [114,115].

### Responsiveness to Emerging Needs

While the current ATN agenda focuses on community and provider interventions that are acceptable to youth and scalable at the national level, the network’s structure is nimble enough to allow for additional high-priority studies generated internally within the network and/or in collaboration with other networks, agencies, and outside investigators. The EHE identified several focus and geographical areas where ATN investigators and research programs can contribute [27]. Taking the EHE focus areas into consideration, the network solicited research studies in 2019 that included a focus on (1) conducting assessments that use an implementation science framework, (2) expanding ongoing efforts in EHE geographic areas to reach underserved youth, or (3) building connections with health departments and other community-based partners in EHE geographic areas.

New projects resulting from this allow the network to increase its youth research portfolio in focused EHE geographic areas where ATN is currently not represented and to contribute to efforts to end the HIV epidemic. The ATN will continue to regularly review its priorities and launch new studies in response to changing needs, scientific advances, and epidemiological shifts in HIV incidence.

## Conclusions

This paper describes the top five research priority areas guiding ATN’s efforts to address the domestic HIV epidemic in youth. Monitoring implementation and progress from current ATN projects requires ongoing monitoring of the indicators of coverage and outcomes as youth progress through the HIV prevention and care continua, and targeting efforts in areas most heavily impacted such as EHE hotspots. Addressing the research agenda and key priority areas for youth at high risk for HIV infection and youth living with HIV also necessitates ongoing collaborations between academic research institutions, health care providers, community-based organizations, impacted communities, and youth in the United States, as well as scientific leadership and expertise on state-of-the-art HIV prevention and care research for adolescents through the ATN.

## Abbreviations

ATN: Adolescent Medicine Trials Network for HIV/AIDS Interventions
ART: antiretroviral therapy
EC: Executive Committee
EHE: Ending the HIV Epidemic: A Plan for America
GBT: gay, bisexual, and transgender
LGBTQ: lesbian, gay, bisexual, transgender, and queer
MSM: men who have sex with men
NIH: National Institutes of Health
PAW: Policy and Advocacy Workgroup
PEP: postexposure prophylaxis
PrEP: pre-exposure prophylaxis
STI: sexually transmitted infection
